# The Cardioprotective Effects of Hydrogen Sulfide in Heart Diseases: From Molecular Mechanisms to Therapeutic Potential

**DOI:** 10.1155/2015/925167

**Published:** 2015-05-11

**Authors:** Yaqi Shen, Zhuqing Shen, Shanshan Luo, Wei Guo, Yi Zhun Zhu

**Affiliations:** ^1^Department of Pharmacology, School of Pharmacy, Fudan University, Zhangheng Road 826, Pudong New District, Shanghai 201203, China; ^2^Department of Physiology and Pathophysiology, Shanghai Medical College, Fudan University, Shanghai 200032, China; ^3^Department of Pharmacology, National University of Singapore, Singapore 117597

## Abstract

Hydrogen sulfide (H_2_S) is now recognized as a third gaseous mediator along with nitric oxide (NO) and carbon monoxide (CO), though it was originally considered as a malodorous and toxic gas. H_2_S is produced endogenously from cysteine by three enzymes in mammalian tissues. An increasing body of evidence suggests the involvement of H_2_S in different physiological and pathological processes. Recent studies have shown that H_2_S has the potential to protect the heart against myocardial infarction, arrhythmia, hypertrophy, fibrosis, ischemia-reperfusion injury, and heart failure. Some mechanisms, such as antioxidative action, preservation of mitochondrial function, reduction of apoptosis, anti-inflammatory responses, angiogenic actions, regulation of ion channel, and interaction with NO, could be responsible for the cardioprotective effect of H_2_S. Although several mechanisms have been identified, there is a need for further research to identify the specific molecular mechanism of cardioprotection in different cardiac diseases. Therefore, insight into the molecular mechanisms underlying H_2_S action in the heart may promote the understanding of pathophysiology of cardiac diseases and lead to new therapeutic targets based on modulation of H_2_S production.

## 1. Introduction

Hydrogen sulfide (H_2_S) has been thought of to be just a toxic gas with a strong odor of rotten eggs for hundreds of years. However, with the advancement of scientific technology over the years, researchers have discovered that H_2_S takes part in a series of physiological and pathological processes in mammals. A pioneering study reported by Abe and Kimura [1] in 1996 determined that H_2_S facilitated the induction of hippocampal long-term potentiation by enhancing the activity of N-methyl-D-aspartate (NMDA) receptors. From then on, scientific interest has grown in the investigation of the function of H_2_S as a gasotransmitter.

Now H_2_S has been regarded as a novel gaseous signaling molecule, similarly to nitric oxide (NO) and carbon monoxide (CO) [2, 3]. H_2_S is endogenously produced by several enzymes, including cystathionine-*β*-synthase (CBS), cystathionine-*γ*-lyase (CSE), and 3-mercaptopyruvate sulfurtransferase (3-MST) along with cysteine aminotransferase (CAT) [4–7]. The distributions of these enzymes' expressions are tissue specific. CBS is the critical enzyme for H_2_S production in the nervous system and CSE is the major H_2_S-producing enzyme in the cardiovascular system [8]. A number of studies have demonstrated that H_2_S may be involved in a multitude of pathophysiologic processes, such as oxidative stress, inflammation, apoptosis, and angiogenesis [3]. In recent years, growing evidence has showed that H_2_S is a critical regulator of heart functions and plays a protective role in the pathogenesis and development of heart diseases.

In this review, we summarize the biosynthesis and physiological functions of H_2_S and explore its emerging pathogenic significance in several heart diseases including myocardial ischemia/reperfusion (I/R) injury, myocardial infarction, arrhythmias, cardiac hypertrophy, cardiac fibrosis, and heart failure. Furthermore, we also discuss the molecular mechanisms involved in the cardioprotective effects of H_2_S and how these might be used therapeutically to overcome some of the heart diseases.

## 2. Biosynthesis and Metabolism of H_**2**_S

**Figure 1:**
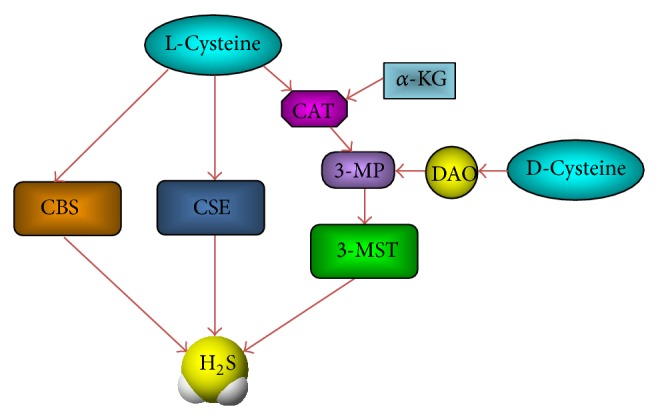
Biosynthesis pathways of endogenous H_2_S. Cystathionine-*β*-synthase (CBS) and cystathionine-*γ*-lyase (CSE) use L-cysteine as a substrate to produce H_2_S. However, 3-mercaptopyruvate sulfurtransferase (3-MST) uses 3-mercaptopyruvate (3-MP) as a substrate to form H_2_S. 3-MP is produced by cysteine aminotransferase (CAT) from L-cysteine in the presence of *α*-keto glutarate (*α*-KG); on the other hand, it is also produced by D-amino acid oxidase (DAO) from D-cysteine.

H_2_S is a small molecule which can pass through cell membranes freely. The basal level of its production in mammalian tissues is determined by the activity of three key enzymes: CBS, CSE, and 3-MST together with CAT (Figure 1). Recent studies have provided a broader picture of enzyme distribution; for example, CBS is expressed in brain, liver, kidney, ileum, uterus, placenta, and pancreatic islets, and it is the predominant producer of H_2_S in the central nervous system [9–11]. CSE is the main H_2_S-generating enzyme in the cardiovascular system and is also found in the liver, kidney, ileum, thoracic aorta, portal vein, uterus, and placenta and is weakly detected in the brain [9, 10, 12, 13]. 3-MST, along with CAT, is a third H_2_S-producing enzyme in neurons, vascular endothelium, and the retina [14–17]. Both CBS and CSE are pyridoxal-5-phosphate- (PLP-) dependent enzymes and located in cytosol; they use L-cysteine as their principal substrate to produce H_2_S [18]. Unlike CBS and CSE, 3-MST and CAT have been found in both mitochondria and cytosol, although approximately two-thirds of 3-MST exists in the mitochondria [19]. 3-MST produces H_2_S from 3-mercaptopyruvate (3MP), which is produced by CAT from L-cysteine and *α*-ketoglutarate [17]. In addition to the above pathway, Kimura group discovered a novel pathway for the production of hydrogen sulfide from D-cysteine in mammalian cells [20]. D-Cysteine is metabolized by d-amino acid oxidase (DAO) to 3MP, which is a substrate for 3-MST to produce H_2_S. This pathway is functional only in the kidney and the brain, particularly in the cerebellum.

H_2_S can undergo several catabolic pathways in order to maintain a proper physiological balance of its metabolism under physiological conditions. Firstly, once deprotonated, HS^−^ is rapidly oxidized in the mitochondria to form thiosulfate (nonenzymatic conversion), followed by further conversion into sulfite and finally into sulfate, the major end product of H_2_S metabolism [21]. Secondly, H_2_S can also be methylated by thiol S-methyltransferase to form dimethylsulfide and methanethiol. Lastly, H_2_S can react with methemoglobin to form sulfhemoglobin [22]. Metabolic labeling studies with Na_2_^35^S have indicated tissue specific differences in sulfide catabolism rates and in product distribution [23]. Rat liver converts sulfide primarily to sulfate, kidney to a mixture of thiosulfate and sulfate, and lung predominantly to thiosulfate. These biosynthetic and degradative pathways for H_2_S will likely prompt more interest into the translational cardioprotective potential of this gasotransmitter in the future.

## 3. Disturbance of Endogenous H_**2**_S Generation in Heart Diseases

The discovery of CSE in the rat heart as well as identification of H_2_S as an important modulator is a breakthrough in the investigation of the role of H_2_S in heart function. Increasing evidence has demonstrated that disturbed H_2_S production is relevant to heart disease. In clinical patients, Jiang et al. [24] found plasma H_2_S levels were significantly lowered in coronary heart disease (CHD) patients compared with that in angiographically normal control subjects. Moreover, in CHD patients, plasma H_2_S levels in unstable angina patients and acute myocardial infarction patients were significantly lower than that in stable angina patients. In addition, Polhemus et al. [25] found that heart failure (HF) patients had marked reductions in circulating H_2_S levels compared to age matched controls. In experimental animal model, studies also show that the endogenous production of H_2_S is significantly reduced in many heart diseases, including myocardial ischemia, myocardial infarction- (MI-) induced or arteriovenous fistula-induced HF, and spontaneous, pulmonary, or hyperhomocysteinemia-induced hypertension [26]. These findings imply that cardiac disease may impair the endogenous synthesis of H_2_S, which may further exacerbate the disease state. Meanwhile, these findings are clear evidence which support the involvement of endogenous H_2_S in maintaining basal physiological functions of the heart.

## 4. Role of H_**2**_S in Heart Diseases

**Figure 2:**
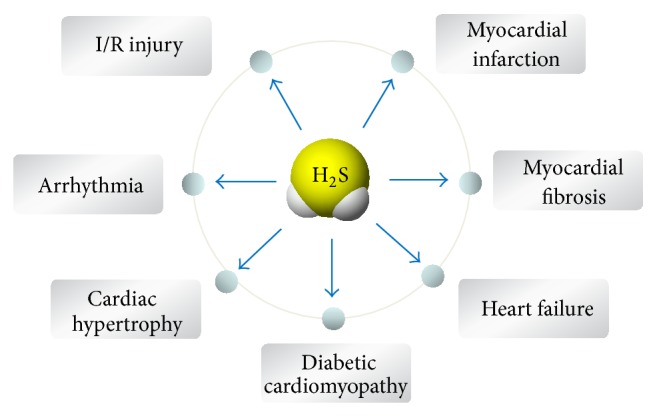
Cardioprotective effects of H_2_S in different heart disease. H_2_S protects the heart against myocardial ischemia/reperfusion injury, myocardial infarction, arrhythmia, myocardial fibrosis, cardiac hypertrophy, heart failure, and diabetic cardiomyopathy.

Recently, H_2_S has been widely recognized as a cardioprotective agent for majority of cardiac disorders. Growing evidence has revealed that H_2_S improves cardiac function and cardiac complications in different pathogenic conditions, such as myocardial I/R injury, myocardial infarction, cardiac arrhythmia, cardiac hypertrophy, myocardial fibrosis, and heart failure (Figure 2).

### 4.1. Myocardial I/R Injury

I/R injury is one critical cause of tissue destruction and often leads to heart failure. Although reperfusion relieves ischemia, it also results in a complex reaction that leads to cell injury caused by inflammation and oxidative damage [27]. A growing body of evidence indicates that H_2_S is involved in myocardial I/R injury. H_2_S postconditioning effectively protects isolated rat hearts against I/R injury via activation of the JAK2/STAT3 signaling pathway, an important component of the survivor activating factor enhancement (SAFE) pathway [28]. In another study, sulfur dioxide (SO_2_) preconditioning can significantly reduce I/R-induced myocardial injury* in vivo*, which is associated with increased myocardial antioxidative capacity and upregulated H_2_S/CSE pathway [29]. H_2_S infusion but not bolus administration markedly reduced myocardial infarct size and improved regional left ventricular function in a porcine I/R model by suppressing cardiomyocyte apoptosis and autophagy [30]. Furthermore, NaHS pretreatment protects isolated rat hearts against I/R injury by inhibition of mitochondria permeability transition pore (MPTP) opening [31]. Our group also found pharmacologic inhibition of CSE resulted in an increase in infarct size in a rat I/R model; conversely, H_2_S replacement displayed myocardial protection [32]. Additionally, cardiac specific CSE overexpressed in transgene mice significantly reduced infarct size and improved cardiac function compared to the wild-type group after 45 minutes of ischemia and 72 hours of reperfusion [33]. These findings reveal that both exogenous donors and endogenously elevated H_2_S serve to protect heart against I/R injury and may serve as an important therapeutic target.

### 4.2. Myocardial Infarction

Myocardial infarction (MI) is the leading cause of death worldwide. It occurs when a coronary artery is occluded, leading to insufficient oxygen supply to the myocardium and resulting in death of cardiomyocytes and nonmyocyte cells [34, 35]. More and more evidence indicates that H_2_S has direct benefits for myocardial infarction. Our group demonstrated for the first time that decreased H_2_S levels in the plasma were associated with an increased infarct size and mortality. NaHS significantly decreased the infarct size of the left ventricle and mortality after acute MI in rats [36]. We also found S-propargyl-cysteine (SPRC), a novel modulator of endogenous hydrogen sulfide, could protect against MI by reducing the deleterious effects of oxidative stress through increased CSE activity and plasma H_2_S concentration [37]. Moreover, we found that increased CSE and H_2_S levels* in vivo* by miR-30 family inhibitor can reduce infarct size, decrease apoptotic cell number in the peri-infarct region, and improve cardiac function in response to MI [38]. Qipshidze et al. [39] also found that administration of H_2_S remarkably ameliorated infarct size and preserved left ventricular function during development of MI in mice. This cardioprotective effect was associated with the improvement of angiogenesis due to inhibition of antiangiogenic proteins and stimulation of angiogenic factors such as vascular endothelial growth factor (VEGF). In another study, Xie et al. [40] found that H_2_S preconditioning effectively promoted mesenchymal stem cells (MSCs) survival under ischemic injury and helped cardiac repair after myocardial infarction in rats.

### 4.3. Cardiac Arrhythmias

Cardiac arrhythmias are an important problem in coronary I/R therapy and constitute a major risk for sudden death after coronary artery occlusion [41]. The primary causes for I/R-induced arrhythmias are considered to be the endogenous metabolites, such as reactive oxygen species (ROS), calcium, thrombin, and platelet activating factor, produced and accumulated in the myocardium during reperfusion.

Zhang et al. [42] found that reperfusion with NaHS after ischemia attenuated arrhythmias in the isolated Langendorff-perfused heart and improved cardiac function during I/R. These effects could be blocked by the ATP-sensitive potassium (K_ATP_) channel blocker glibenclamide, indicating that the cardioprotective effect of H_2_S against arrhythmias during reperfusion at least partially depends on the opening of K_ATP_ channel. Bian et al. [43] also found that blockade of endogenous H_2_S synthesis increased both the duration of I/R-induced arrhythmias and the severity of the arrhythmias. However, preconditioning with 100 *μ*M NaHS attenuated arrhythmias in the isolated heart, increased cell viability, and improved cell function in cardiac myocytes during I/R, and these effects may be mediated by protein kinase C (PKC) and sarcolemmal K_ATP_ channels. Connexin 43 (Cx43) is the principal connexin in the mammalian ventricle and has been proven to have a close association with arrhythmia [44]. Huang et al. [45] found that H_2_S ameliorated the expression of Cx43 in cardiac tissue, which indicated that endogenous H_2_S may play an important role in regulating heart function and arrhythmia. Furthermore, Yong et al. [46] found that lowered H_2_S production during ischemia may cause overstimulation of the *β*-adrenergic function which was closely linked with the incidence of ventricular arrhythmias. Exogenous application of H_2_S negatively modulated *β*-adrenergic function by inhibiting adenylyl cyclase activity and finally protected heart against cardiac arrhythmias.

Based on these findings, H_2_S replacement therapy may be a significant cardioprotective and antiarrhythmic intervention for those patients with chronic ischemic heart disease whose plasma H_2_S level is reduced.

### 4.4. Myocardial Fibrosis

Cardiac fibrosis is characterized by net accumulation of extracellular matrix proteins in the cardiac interstitium and contributes to both systolic and diastolic dysfunction in many processes of cardiac disorders [47]. Although the fibroblast activation and proliferation are important for maintaining cardiac integrity and function early after cardiac injury, the development of fibrous scar tissue in the infarct zone often leads to chronic complications and functional insufficiencies [48].

Mishra et al. [49] found cardiac fibrosis and apoptosis in chronic heart failure (CHF) were reversed by administration of H_2_S, which was associated with a decrease in oxidative and proteolytic stresses. In addition, Huang et al. [45] revealed that H_2_S markedly prevented the development of cardiac fibrosis and decreased the collagen content in the cardiac tissue by inhibiting the activity of intracardiac Ang-II. It is well known that multiple potassium channels are expressed in cardiac ventricular fibroblasts [50], whereby their modulations may have major significance in cardiac fibrosis. Sheng et al. [51] found that H_2_S potentially modulate cardiac fibrosis by inhibiting large conductance Ca^2+^-activated K^+^ current (BK_Ca_), transient outward K^+^ current (Ito), and Ba^2+^-sensitive inward rectifier K^+^ current (IK_ir_), independent of K_ATP_ channels, leading to decreased proliferation and suppression of transforming growth factor-*β*1- (TGF-*β*1-) induced myofibroblast transformation of atrial fibroblasts. Our previous finding has demonstrated that H_2_S therapy significantly attenuated ischemia-induced cardiac fibrosis in chronic heart failure rats [52]. We also found that treatment with H_2_S substantially inhibited AngII-stimulated cardiac fibroblasts, as evidenced by the reduction in *α*-SMA and type I collagen expression as well as effective suppression of the fibrotic marker CTGF. In addition, we proved that the pharmacologic supplementation of exogenous H_2_S attenuated fibrotic and inflammatory responses induced by MI. The beneficial effects of H_2_S, at least in part, were associated with a decrease of Nox4-ROS-ERK1/2 signaling axis and an increase in heme oxygenase-1 (HO-1) expression [53].

### 4.5. Cardiac Hypertrophy

Cardiac hypertrophy, usually considered as an effective compensation mechanism, can maintain or even increase cardiac output. However, in the long term, persistent hypertrophy will ultimately result in cardiac dilatation, decreased ejection fraction, and subsequent heart failure [54]. Pathological hypertrophy usually occurs in response to chronically increased pressure overload or volume overload, or following MI.

A large number of experiments confirm that H_2_S play a positive role in protecting heart against cardiac hypertrophy. Lu et al. [55] demonstrated that H_2_S could improve cardiac function and reduce myocardial apoptosis in the isoproterenol- (ISO-) induced hypertrophy rat model by reducing Nox4 expression and ROS production in the mitochondria. Treatment of mice with sodium sulfide (Na_2_S) leads to less cardiac hypertrophy and left ventricular dilatation as well as improved left ventricular function after the induction of heart failure in a thioredoxin 1- (Trx1-) dependent manner [56]. In addition, pharmacologic H_2_S therapy during heart failure serves to mitigate pathological left ventricular remodeling and reduce myocardial hypertrophy, oxidative stress, and apoptosis [49]. In an endothelin-induced cardiac hypertrophy rat model, Yang et al. [57] found that H_2_S treatment could decrease left ventricular mass index, volume fraction of myocardial interstitial collagen, and myocardial collagen content and improve cardiac hypertrophy. In another hypertrophy model induced by abdominal aorta coarctation, Huang et al. [58] revealed that exogenous administration of H_2_S significantly suppressed the development of cardiac hypertrophy and also greatly downregulated the Ang-II levels in cardiac tissue, suggesting that H_2_S plays a pivotal role in the development of pressure overload-induced cardiac hypertrophy. Interestingly, Padiya et al. [59] showed that administration of freshly prepared homogenate of garlic, which have been shown to generate H_2_S after interaction within cellular proteins, can activate myocardial nuclear-factor-E2-related factor-2 (Nrf2) through PI3K/AKT pathway and attenuate cardiac hypertrophy and oxidative stress through augmentation of antioxidant defense system in fructose-fed insulin resistance rats.

## 5. Heart Failure

Heart failure (HF) is a heterogeneous syndrome that can result from a number of common disease stimuli, including long-standing hypertension, myocardial infarction, or ischemia associated with coronary artery disease. The pathogenesis of HF has not been fully elucidated and the current treatments for HF are woefully inadequate. H_2_S therapy has recently been shown to ameliorate ischemic-induced heart failure in a murine model. Cardiac-restricted overexpression of CSE in mice resulted in increased endogenous H_2_S production and a profound protection against ischemia-induced heart failure and decreased mortality [60]. In contrast, knockout of CSE in murine models of heart failure showed worsened myocardial function and greater infarct size [61].

In a hypertension-induced heart failure model, it has been demonstrated clearly that H_2_S decelerated progression to adverse remodeling of the left ventricle and induced angiogenesis in the myocardium [62]. Polhemus et al. [63] also found H_2_S therapy attenuated left ventricular remodeling and dysfunction in the setting of heart failure by creating a proangiogenic environment for the growth of new vessels. In another model of pressure overload-induced heart failure, mice administered Na_2_S exhibited enhanced proangiogenesis factors, such as matrix metalloproteinase- (MMP-) 2, and suppressed antiangiogenesis factors, including MMP-9 [64]. H_2_S also play a protective role in volume overload-induced CHF by upregulating protein and mRNA expression of HO-1 [65].

Local cardiac renin-angiotensin system (RAS) is required for the development of heart failure and left ventricular remodeling. Liu and coworkers [66] have demonstrated that treatment with NaHS could protect against isoproterenol-induced heart failure by suppression of local renin levels through inhibition of both mast cell infiltration and renin degranulation in rats, suggesting a novel mechanism for H_2_S-mediated cardioprotection against heart failure. Our group found NaHS markedly inhibited cardiac apoptosis and improved mitochondrial derangements, both of which led to cardioprotection in a rat model of heart failure [52]. In addition, we also showed that NaHS decreased the leakage of cytochrome c protein from the mitochondria to the cytoplasm, improved mitochondrial derangements, and increased CSE mRNA and protein levels in heart failure rats [52]. SPRC, reported also as ZYZ-802, could reduce infarct size and improve cardiac function in a rat model of MI-induced heart failure via antiapoptosis and antioxidant effects as well as angiogenesis promotion [67, 68]. All these illustrate that the CSE/H_2_S pathway plays a critical role in the preservation of cardiac function in heart failure.

### 5.1. Diabetic Cardiomyopathy

Diabetic cardiomyopathy (DCM) is a distinct primary disease process which occurs independently of coronary artery disease and hypertension, resulting in structural and functional abnormalities of the myocardium leading to HF [69]. Increasing evidence has proved that H_2_S plays a positive role in regulating diabetic myocardial injury.

A current study [70] showed that both plasma H_2_S levels and plasma H_2_S synthesis activity were significantly reduced in the streptozotocin- (STZ-) induced diabetic rats. In addition, H_2_S was also decreased in the plasma of type 2 diabetic patients compared with age matched healthy controls [71]. These findings suggest the involvement of H_2_S in diabetic pathological processes. Xu et al. [72] found exogenous H_2_S exerted a protective effect against high glucose- (HG-) induced injury by inhibiting the activation of the p38 MAPK and ERK1/2 pathways and preventing oxidative stress in H_9_C_2_ cells. Wei et al. [73] also reported that a novel H_2_S-releasing molecule GYY4137 probably protected H_9_C_2_ cells against HG-induced cytotoxicity by activation of the AMPK/mTOR signal pathway. Moreover, H_2_S may reduce HG-induced oxidative stress by activating Nrf2/ARE pathway and may exert antiapoptotic effects in diabetic myocardium by inhibiting JNK and p38 MAPK pathways and activating PI3K/Akt signaling [74]. Interestingly, Padiya et al.'s study [59] showed that administration of raw garlic homogenate in insulin resistance fructose fed rat activated myocardial Nrf2 by increasing H_2_S level and activating PI3K/AKT pathway and attenuated cardiac hypertrophy and oxidative stress through augmentation of antioxidant defense system. In another study, using a STZ-induced diabetes model in rats, Zhou et al. [74] demonstrated an important therapeutic potential of the H_2_S pathway in DCM. They found that daily administration of NaHS had anti-inflammatory, antioxidative, and antiapoptotic effects and rescued the decline in heart function in the STZ + NaHS group. Furthermore, Peake et al. [75] found that exogenous administration of Na_2_S attenuated myocardial I/R injury in db/db mice, suggesting the potential therapeutic effects of H_2_S in treating a heart attack in the setting of type 2 diabetes.

## 6. Molecular Mechanisms of H_**2**_S-Induced Cardioprotection

**Figure 3:**
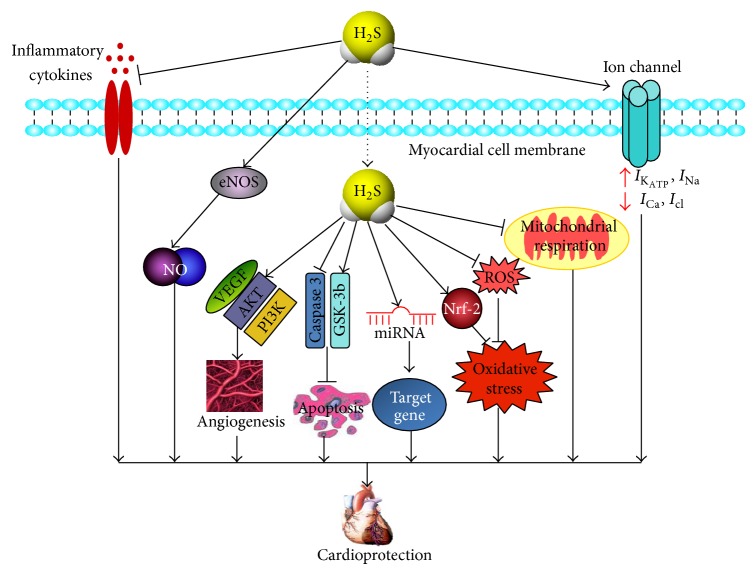
Different signaling pathways activated by H_2_S showing the cardioprotective effects. H_2_S can protect heart against diseases via different mechanisms: H_2_S prevents inflammatory response mediated by inflammatory cytokines. H_2_S stimulates angiogenesis by increasing the expression of VEGF and activating phosphatidylinositol 3-kinase (PI3K) and Akt. H_2_S activates endothelial nitric oxide synthase (eNOS) and augments NO bioavailability. H_2_S significantly protects against cardiomyocyte apoptosis by suppressing the activation of caspase-3 and upregulating the expression of glycogen synthase kinase-3 (GSK-3*β*). H_2_S plays its role by regulating the expression of miRNA. H_2_S also protects mitochondrial function via inhibition of mitochondrial respiration. H_2_S exerts antioxidative action by activating nuclear-factor-E2-related factor-2 (Nrf2) dependent pathway and scavenging of ROS. H_2_S opens K_ATP_ channels, increases Na^+^ channels (Nav) current, and inhibits L-type Ca^2+^ channels and chloride channels, to produce cardioprotective effects.

Similar to NO and CO, the effects of H_2_S on the heart are mediated via a diverse array of cellular and molecular signals. The mechanisms by which H_2_S protects against cardiac diseases are through antioxidative action, preservation of mitochondrial function, reduction of cardiomyocyte apoptosis, anti-inflammatory responses, angiogenic action, regulation of ion channel, and increasing the production of NO (Figure 3).

### 6.1. Antioxidative Action

Oxidative stress is a process due to an imbalance between prooxidant and antioxidant systems. Oxidative stress-induced cellular injury is often caused by excessive formation of ROS, such as superoxide anion (O^2−^), hydroxyl radical (OH^−^), peroxynitrite (ONOO^−^), and hydrogen peroxide (H_2_O_2_). The occurrence of the majority heart diseases is associated with ROS generation, including myocardial I/R injury, cardiac hypertrophy, myocardial fibrosis, and arrhythmias. H_2_S has been reported as a strong antioxidant and widely proposed to protect the cardiac system through its antioxidant role. The robust antioxidant actions of H_2_S are associated with direct scavenging of ROS and/or increased expressions and functions of antioxidant enzymes.

Sun et al. [76] found that H_2_S inhibited mitochondrial complex IV activity and increased the activities of Mn-SOD and CuZn-SOD and decreased the levels of ROS in cardiomyocytes during I/R. H_2_S decreased lipid peroxidation by scavenging hydrogen peroxide and superoxide in a model of isoproterenol-induced myocardial injury [77]. The activation of Nrf2 dependent pathway mediated by H_2_S results in upregulated gene expression of specific factors, such as HO-1, gluthatione reductase, glutathione S-transferase, thioredoxin, and catalase, which play role in endogenous antioxidant defense. Furthermore, H_2_S has an inhibitory effect on phosphodiesterase-5 (PDE-5), which results in decreased NADPH oxidase formation, and the level of antioxidant enzymes increases [78]. Besides these mechanisms, H_2_S also acts as a direct scavenger to neutralize cytotoxic reactive species like peroxynitrite [79] and directly destroys organic hydroperoxides of pathobiological importance, like fatty acid hydroperoxides (LOOHs) [80]. Collectively, these findings suggest that H_2_S is capable of preventing the generation of ROS, scavenging ROS, and strengthening the endogenous antioxidant system.

### 6.2. Preservation of Mitochondrial Function

Mitochondrial function is compromised under hypoxic conditions or in the presence of increased ROS [81]. Growing evidence has shown that H_2_S has the ability to protect mitochondria and ultimately improve respiration and promote biogenesis. Elrod and colleagues [33] found a dose dependent reduction of oxygen consumption in isolated murine cardiac mitochondria after hypoxia, and the administration of H_2_S was shown to improve the recovery of posthypoxic respiration rate significantly. Moreover, electron microscopy showed a notable reduction in mitochondrial swelling and increased matrix density in mice after treatment with H_2_S, further suggesting a prominent role of H_2_S in the preservation of mitochondrial function in the cytoprotection. In addition, H_2_S can affect mitochondria of cardiac cells by inhibition of cytochrome c oxidase in a potent and reversible way, which leads to preservation of mitochondrial structure and function [52]. H_2_S may protect mitochondrial function by inhibiting respiration, thus limiting the generation of ROS and diminishing the degree of mitochondrial uncoupling, leading to decreased infarct size and preserved function [33]. Furthermore, H_2_S preserved mitochondrial function after reperfusion as noted by increased complex I and II efficiency, leading to downregulated mitochondrial respiration and subsequent cardioprotective effects during myocardial I/R injury [82]. Downregulation of MPTP can reduce mitochondrial membrane potential depolarization and consequently inhibit the activation of proapoptotic protein [83]. It is reported that H_2_S can affect mitochondrial targets via upregulation of the reperfusion injury salvage kinase pathway, which is able to inhibit the opening of mitochondria permeability transition pores (MPTP) [84].

### 6.3. Antiapoptosis

There is increasing proof that H_2_S has antiapoptotic actions. Most data indicate the antiapoptotic effects of H_2_S are mainly due to the preservation of mitochondrial function, and many of the cytoprotective actions of H_2_S during ischemic states may be a result of potent actions on mitochondria [85]. It is reported that H_2_S significantly protected against high glucose-induced cardiomyocyte apoptosis by altering Bax and Bcl-2 gene expression [86]. Moreover, It is found that NaHS treatment suppressed the activation of caspase-3 and reduced apoptotic cell numbers in both mice [33] and swine [87], suggesting that H_2_S was capable of inhibiting the progression of apoptosis after I/R injury.

Survivin is an antiapoptotic gene implicated in the initiation of mitochondrial-dependent apoptosis. In an* in vivo* I/R rat model, our group found administration of NaHS for 6 days before surgery significantly upregulated survivin mRNA and protein expressions by 3.4-fold and 1.7-fold, respectively [32], suggesting another way of action for H_2_S-induced cardioprotection.

The activity of glycogen synthase kinase-3 (GSK-3*β*), which has been proposed as a viable target in the ischemic heart injury, is associated with both apoptosis and cell survival. Osipov et al. [30] found that H_2_S infusion increased the expression of the phosphorylated form of GSK-3*β* significantly. Similarly, Yao et al. [88] also demonstrated that NaHS upregulated the phosphorylation of GSK-3*β* (Ser9) expression and subsequently resulted in inhibiting the opening of MPTP, preventing apoptosis and protecting the heart against ischemic damage.

### 6.4. Anti-Inflammation

Inflammation is involved in the main pathological processes of ischemic heart disease. For example, cytokines mediate the development of ischemic injury in the heart and depress myocardial function [89]. IL-6 and IL-8 are released on myocardial I/R damage and then increase neutrophil adhesion and inflammatory responses [90]. TNF-*α* plays multiple roles in the pathogenesis of myocardial I/R injury by inducing endothelium adhesion molecules, allowing for neutrophil infiltration, increasing the production of ROS, amplifying the inflammatory response, and having direct myocardial depressant and apoptotic actions [91].

Studies have shown that H_2_S may play dual roles in inflammatory process. Whiteman and Winyard [92] reviewed 14 studies showing an anti-inflammatory effect of H_2_S and 15 studies showing a proinflammatory effect of H_2_S. However, the anti-inflammatory effect of H_2_S plays a dominant role in heart disease. In myocardial I/R experiments, Elrod et al. [33] have demonstrated that, at the time of heart reperfusion, H_2_S decreased the number of leukocytes within the ischemic zone as well as neutrophils within the myocardial tissue. The evaluation of inflammatory cytokines revealed myocardial levels of IL-1*β* to be markedly reduced after administration of H_2_S. Additionally, H_2_S was found to potently reduce* in vivo* leukocyte-endothelial cell interactions. Using the ischemic porcine heart, Sodha et al. [93] found that NaHS treatment decreased the level of TNF-a, IL-6, and IL-8 as well as the activity of myeloperoxidase. Therefore, H_2_S restrained the extent of inflammation and limited the extent of MI by preventing leukocyte transmigration and cytokine release. In another study, the H_2_S donor, Na_2_S and NaHS were both able to inhibit leukocyte adherence and the resultant inflammatory pathology via activation of K_ATP_ channels [94].

In the lipopolysaccharide-induced inflammatory response of rat embryonic ventricular myocardial cells (H_9_C_2_ cells), our group also found [95] that SPRC prevented nuclear factor-*κ*B (NF-*κ*B) activation and suppressed LPS-induced extracellular signal-regulated kinase 1/2 (ERK1/2) phosphorylation and intracellular reactive oxygen species (ROS) production. In addition, SPRC induced phosphorylation of Akt, attenuated LPS-induced mRNA and protein expression of tumor necrosis factor-*α* (TNF-*α*), and inhibited mRNA expression of intercellular adhesion molecule-1 (ICAM-1) and inducible nitric oxide synthase (iNOS). Therefore, SPRC produced an anti-inflammatory effect in LPS-stimulated H_9_C_2_ cells through the CSE/H_2_S pathway by impairing I*κ*B*α*/NF-*κ*B signaling and by activating PI3K/Akt signaling pathway. These studies provide strong evidence of the function of H_2_S as anti-inflammatory agent.

### 6.5. Angiogenesis

The cardioprotective role of H_2_S could also be due to its angiogenic action on the ischemic area in the heart. Angiogenesis plays a pivotal role in the early stage of wound healing. In* in vitro* studies, incubation with low micromolar concentrations of H_2_S increased endothelial cell number, cell migration, and capillary morphogenesis on matrigel [96]. Chicken chorioallantoic membranes, an* in vivo* model of angiogenesis, displayed increased branching and lengthening of blood vessels in response to 48 h treatment with H_2_S [97]. Aortic rings isolated from CSE knockout mice exhibited markedly reduced microvessel formation. Additionally, in a wound healing model, topically applied H_2_S accelerated wound closure and healing [97].

Angiogenesis is very important in chronic ischemia as poorly vascularized tissue will result in loss of function. Therefore, increasing myocardial vascularity and perfusion in concert with cardiac myocyte growth are critical to prevent the progression of heart failure. In a hypertension-induced heart failure model, administration of H_2_S induced angiogenesis in the myocardium and decelerated the progression of left ventricle remodeling [63]. In a similar heart failure model, NaHS treatment improved cardiac function and mitigated transition from compensatory hypertrophy to heart failure, which was associated with a significant increase in capillary density [98]. In another MI model, H_2_S supplementation showed improvement of heart function and mitigation of cardiac remodeling by increasing angiogenic vessels and blood flow in MI mice [39].

Multiple signaling mechanisms are involved in the angiogenic action of H_2_S, including activation of K_ATP_ channels [99]. By using the K_ATP_ channel inhibitor glibenclamide, Papapetropoulos et al. [97] found that K_ATP_ channel was involved in H_2_S-stimulated angiogenesis. Additionally, H_2_S can stimulate angiogenesis through phosphatidylinositol 3-kinase (PI3K) and Akt activation [96]. H_2_S can also activate hypoxia inducible factor-1a (HIF-1a) and thus increase expression of VEGF [100]. VEGF is a key growth factor in physiological angiogenesis and induces angiogenesis in myocardial ischemia and MI. H_2_S is reported to promote angiogenesis in a MI model by increasing the expression of VEGF and its specific receptors such as the tyrosine kinase receptor-flk-1 and the fms-like tyrosine kinase-flt-1 [39]. It is also reported that H_2_S can regulate the matrix metalloproteinase/tissue inhibitor of metalloproteinase (MMP/TIMP) axis to promote VEGF synthesis and angiogenesis [98]. Furthermore, Zhu group identified VEGFR2 as a receptor for H_2_S for inducing angiogenesis in vascular endothelial cells and found that an intrinsic inhibitory Cys1045–Cys1024 disulfide bond acted as a molecular switch for H_2_S to regulate the structure and function of VEGFR2. VEGFR2 was directly activated by H_2_S suggesting that VEGFR2 acted as a direct target molecule for H_2_S in vascular endothelial cells [101].

### 6.6. Regulation of Ion Channel

The effects of H_2_S on heart electrophysiology have been reported. There are two different types of Ca^2+^ channels (L-type and T-type) in the myocardial membrane. L-type Ca^2+^ channels are absolutely essential for maintaining the electrophysiological basis for the plateau phase of action potentials and for excitation-contraction (EC) coupling [102]. Whole patch clamp experiments in rat cardiomyocytes revealed that NaHS negatively modulates L-type Ca^2+^ channels composed by the CaV1.2 subunits in rat cardiomyocytes [103–105]. T-type Ca^2+^ channels can be reexpressed in atrial and ventricular myocytes in a variety of pathological conditions such as cardiac hypertrophy and heart failure and participate in abnormal electrical activity and EC coupling [106]. A recent report has showed that NaHS (10 *μ*M–1 mM) selectively inhibits Cav3.2 T-type Ca^2+^ channels which are heterologously expressed in HEK293 cells [107].

K_ATP_ channels are located on the surface of cell membranes and mitochondria and are widely distributed in the myocardium. The opening of K_ATP_ channels is an important endogenous cardioprotective mechanism involved in cardiac ischemia preconditioning. The K_ATP_ channel opening generates outward currents and causes hyperpolarization, which reduces calcium influx via L-type Ca^2+^ channels and prevents Ca^2+^ overload. Tang and coworkers [108] found evidence that NaHS (100 *μ*M) opened the K_ATP_ channels in vascular smooth muscle cells. Furthermore, H_2_S may also indirectly activate the K_ATP_ channels by inducing intracellular acidosis [109]. By activation of the K_ATP_ channels, H_2_S shortens action potential duration (APD) and produces cardioprotective effects [110, 111], though H_2_S has no significant effect on the amplitude of action potential and resting potential [104].

Study has demonstrated that voltage-dependent Na^+^ channels (Nav) can be regulated by H_2_S. In Native Nav from jejunum smooth muscle and recombinant Nav (Nav1.5) heterologously expressed in HEK293, Strege et al. [112] found NaHS increased peak sodium currents and also right-shifted the voltage dependence of Na^+^ current inactivation and activation. This effect could extend beyond the jejunum, since Nav1.5 is also expressed in other tissues. In the heart, Nav1.5 gives rise to the upstroke of the cardiac action potential; thus, it is possible that H_2_S may have the same effect on the Nav expressed in the heart.

Growing studies show that chloride channels play an important role in normal physiological function in myocardial cells, but abnormal changes can be found in pathological conditions such as myocardial ischemia and arrhythmias. Malekova et al. [113] investigated the effect of H_2_S on single-channel currents of chloride channels using the patch clamp technique and found that NaHS inhibited the chloride channels by decreasing the channel open probability in a concentration dependent manner. The inhibitory effect of H_2_S on the chloride channels may be involved in the biological actions of H_2_S in the heart.

### 6.7. Interaction with NO

H_2_S protects cardiac muscles from I/R injury by increasing the production of NO [114]. H_2_S is known to interact with the other biological mediators and signal transduction components to produce its effects in the cardiovascular system. H_2_S can activate endothelial nitric oxide synthase (eNOS) through phosphorylation at the S1177 active site and augment NO bioavailability [61], highlighting that there is an interaction between NO and H_2_S of physiological significance. There is evidence that NO and peroxynitrite react with H_2_S to form a novel nitrosothiol, which has been proposed to regulate the physiological effects of both NO and H_2_S [115]. Moreover, mice treated with the H_2_S donor, diallyl trisulfide (DATS), showed marked increases in plasma nitrite, nitrate, and nitrosylated protein (RXNO) levels 30 minutes after injection [116].

In CSE knockout mice, the levels of H_2_S and bound sulfane sulfur in tissues and blood as well as the levels of NO metabolites were decreased significantly. However, administration of H_2_S rescued the heart form I/R injury by activating eNOS and increasing NO availability. In addition to these observations in CSE knockout mice, the administration of H_2_S failed to protect the cardiac muscle from I/R injury in eNOS defective mutant mice [114]. Similar results were also obtained by Kondo et al. [61] in a mouse model of pressure overload-induced heart failure, which suggests that H_2_S protects the heart by upregulating eNOS phosphorylation accompanied by increasing NO production. Interestingly, plasma H_2_S levels, CSE gene enzymatic activity, and expression in the cardiovascular system were reduced in rats after treated with a NOS inhibitor chronically, indicating the physiological significance of NO in the regulation of H_2_S production in the cardiovascular system [117].

### 6.8. Regulation of miRNA Expression

MicroRNAs (miRNAs) are evolutionarily conserved molecules that modulate the expression of their target genes by mRNA degradation or translational repression, and they may participate in various physiological and pathological processes of heart diseases [118]. An increasing body of evidence shows that H_2_S exerts its role by regulating the expression of miRNA. Shen et al. [119] found H_2_S was involved in regulating the expression of drought associated miRNAs such as miR-167, miR-393, miR-396, and miR-398 and their target genes, and therefore improved the tolerance of Arabidopsis to drought. A recent study [120] demonstrated that H_2_S played a role in the protection of hepatic I/R injury in the young rats by downregulating the expression of miR-34a, which resulted in the promotion of Nrf-2 signaling pathway. More importantly, Liu et al. [121] found H_2_S inhibited cardiomyocyte hypertrophy by upregulating miR-133a. In addition, H_2_S donor, Na_2_S, would attenuate myocardial injury through upregulation of protective miR-21 and suppression of the inflammasome, a macromolecular structure that amplifies inflammation and mediates further injury [122]. These data suggest a new mechanism for the role of H_2_S and indicate that miRNA could be a new target of H_2_S in cardiac disorders.

## 7. H_**2**_S-Based Therapeutic Potential for Heart Diseases

More and more H_2_S donors with varying chemical and pharmacological properties have been reported as potential therapeutics. Among them, Na_2_S and NaHS were the first H_2_S-releasing agents studied in the cardiac system [33, 123]. As inorganic salts, Na_2_S and NaHS have the advantage of rapidly increasing H_2_S concentration within seconds, but they also rapidly decline within tissue and could exert adverse side effects because of rapid increases in H_2_S at high concentrations [124]. This somewhat limits their therapeutic potential. Thus, it is important to develop novel H_2_S-releasing drugs used to treat heart diseases.

Synthetic H_2_S-releasing compounds have been developed. GYY4137, a water-soluble compound capable of releasing H_2_S slowly, has been reported to protect against high glucose-induced cytotoxicity by activation of the AMPK/mTOR signal pathway in H_9_C_2_ cells [73]. SG-1002 [61] and penicillamine based donors [125] are examples of synthesized H_2_S donors whose release is more precisely controlled. H_2_S therapy with SG-1002 resulted in cardioprotection in the setting of pressure overload-induced heart failure via upregulation of the VEGF-Akt-eNOS-NO-cyclic guanosine monophosphate (cGMP) pathway with preserved mitochondrial function, attenuated oxidative stress, and increased myocardial vascular density. Penicillamine based donors showed potent protective effects in an* in vivo* murine model of myocardial I/R injury.

In recent years, some natural plant-derived compounds, such as garlic, have been found to produce H_2_S. Naturally occurring H_2_S donors such as DATS, a polysulfide derived from garlic, is known to protect against myocardial I/R injury in mice through preservation of endogenous H_2_S [126]. It also has been shown to protect against hyperglycemia-induced ROS-mediated apoptosis by upregulating the PI3 K/Akt/Nrf2 pathway, which further activates Nrf2-regulated antioxidant enzymes in cardiomyocytes exposed to high glucose [127]. Additionally, organic sulfide donors derived from garlic, such as diallyl disulfide (DADS), attenuate the deleterious effects of oxidized LDL on NO production [128] and protect the ischemic myocardium. SAC (S-allylcysteine), another derivative of garlic, significantly lowers mortality and reduces infarct size following MI [129]. SPRC, a structural analogue of SAC which was synthesized by our group, was found to protect against myocardial ischemic injury both in* in vivo* and* in vitro* studies through the increase in CSE activity and plasma H_2_S concentration [130]. SAC and SPRC are both cardioprotective in MI by modulating the endogenous levels of H_2_S, reducing the deleterious effects of oxidative stress and preserving the activities of antioxidant-defensive enzymes like SOD [37]. As novel H_2_S releasing agents or H_2_S donors develop, these novel agents should ultimately address the clinically relevant issues such as sustained release or half-life, route of administration, tissue specificity, and low toxicity.

## 8. Conclusion and Perspectives

Following in the footsteps of NO and CO, H_2_S is rapidly emerging as a critical cardiovascular signaling molecule. We have summarized the current knowledge on the function of H_2_S in heart disease and discussed the possible molecular mechanisms involved in its cardioprotective effect. Although the complete actions of this gas remain under investigation and the underlying mechanisms should be further elucidated, the therapeutic options relating to heart disease are extremely promising. We also reviewed the current H_2_S donors which have been verified to have the therapeutic potential for heart disorders. Most of the current H_2_S donors have the drawback of rapid degradation and difficult to control. Furthermore, whether the therapeutic effects of these donors in animal studies can be transferable to clinical studies needs to be determined. However, we believe a long-acting donor with controlled H_2_S release will be developed. In short, a better understanding of the function of the H_2_S in heart disease as well as development of novel H_2_S-based therapeutic agents may be helpful to reduce the risks of heart disease in the future.

## References

[1] Abe K., Kimura H. (1996). The possible role of hydrogen sulfide as an endogenous neuromodulator. *The Journal of Neuroscience*.

[2] Wang R. (2002). Two’s company, three’s a crowd: can H_2_S be the third endogenous gaseous transmitter?. *The FASEB Journal*.

[3] Wang R. (2012). Physiological implications of hydrogen sulfide: a whiff exploration that blossomed. *Physiological Reviews*.

[4] Liu H., Bai X.-B., Shi S., Cao Y.-X. (2009). Hydrogen sulfide protects from intestinal ischaemia-reperfusion injury in rats. *Journal of Pharmacy and Pharmacology*.

[5] Wagner F., Asfar P., Calzia E., Radermacher P., Szabó C. (2009). Bench-to-bedside review: hydrogen sulfide—the third gaseous transmitter: applications for critical care. *Critical Care*.

[6] Shibuya N., Tanaka M., Yoshida M. (2009). 3-Mercaptopyruvate sulfurtransferase produces hydrogen sulfide and bound sulfane sulfur in the brain. *Antioxidants and Redox Signaling*.

[7] Yang G., Wu L., Jiang B. (2008). H_2_S as a physiologic vasorelaxant: hypertension in mice with deletion of cystathionine *γ*-lyase. *Science*.

[8] Yang C., Yang Z., Zhang M. (2011). Hydrogen sulfide protects against chemical hypoxia-induced cytotoxicity and inflammation in hacat cells through inhibition of ROS/NF-*κ*B/COX-2 pathway. *PLoS ONE*.

[9] Kaneko Y., Kimura Y., Kimura H., Niki I. (2006). l-cysteine inhibits insulin release from the pancreatic *α*-cell: possible involvement of metabolic production of hydrogen sulfide, a novel gasotransmitter. *Diabetes*.

[10] Patel P., Vatish M., Heptinstall J., Wang R., Carson R. J. (2009). The endogenous production of hydrogen sulphide in intrauterine tissues. *Reproductive Biology and Endocrinology*.

[11] Stipanuk M. H., Beck P. W. (1982). Characterization of the enzymic capacity for cysteine desulphhydration in liver and kidney of the rat. *Biochemical Journal*.

[12] Hosoki R., Matsuki N., Kimura H. (1997). The possible role of hydrogen sulfide as an endogenous smooth muscle relaxant in synergy with nitric oxide. *Biochemical and Biophysical Research Communications*.

[13] Yang W., Yang G., Jia X., Wu L., Wang R. (2005). Activation of K_ATP_ channels by H_2_S in rat insulin-secreting cells and the underlying mechanisms. *The Journal of Physiology*.

[14] Mikami Y., Shibuya N., Kimura Y., Nagahara N., Ogasawara Y., Kimura H. (2011). Thioredoxin and dihydrolipoic acid are required for 3-mercaptopyruvate sulfurtransferase to produce hydrogen sulfide. *Biochemical Journal*.

[15] Mikami Y., Shibuya N., Kimura Y., Nagahara N., Yamada M., Kimura H. (2011). Hydrogen sulfide protects the retina from light-induced degeneration by the modulation of Ca^2+^ influx. *The Journal of Biological Chemistry*.

[16] Shibuya N., Mikami Y., Kimura Y., Nagahara N., Kimura H. (2009). Vascular endothelium expresses 3-mercaptopyruvate sulfurtransferase and produces hydrogen sulfide. *The Journal of Biochemistry*.

[17] Shibuya N., Tanaka M., Yoshida M. (2009). 3-Mercaptopyruvate sulfurtransferase produces hydrogen sulfide and bound sulfane sulfur in the brain. *Antioxidants and Redox Signaling*.

[18] Li L., Rose P., Moore P. K. (2011). Hydrogen sulfide and cell signaling. *Annual Review of Pharmacology and Toxicology*.

[19] Lavu M., Bhushan S., Lefer D. J. (2011). Hydrogen sulfide-mediated cardioprotection: mechanisms and therapeutic potential. *Clinical Science*.

[20] Shibuya N., Koike S., Tanaka M. (2013). A novel pathway for the production of hydrogen sulfide from D-cysteine in mammalian cells. *Nature Communications*.

[21] Kimura H. (2012). Metabolic turnover of hydrogen sulfide. *Frontiers in Physiology*.

[22] Kimura H. (2013). Physiological role of hydrogen sulfide and polysulfide in the central nervous system. *Neurochemistry International*.

[23] Bartholomew T. C., Powell G. M., Dodgson K. S., Curtis C. G. (1980). Oxidation of sodium sulphide by rat liver, lungs and kidney. *Biochemical Pharmacology*.

[24] Jiang H.-L., Wu H.-C., Li Z.-L., Geng B., Tang C.-S. (2005). Changes of the new gaseous transmitter H_2_S in patients with coronary heart disease. *Academic Journal of the First Medical College of PLA*.

[25] Polhemus D. J., Calvert J. W., Butler J., Lefer D. J. (2014). The cardioprotective actions of hydrogen sulfide in acute myocardial infarction and heart failure. *Scientifica*.

[26] Liu Y. H., Lu M., Hu L. F., Wong P. T. H., Webb G. D., Bian J. S. (2012). Hydrogen sulfide in the mammalian cardiovascular system. *Antioxidants & Redox Signaling*.

[27] Dhalla N. S., Elmoselhi A. B., Hata T., Makino N. (2000). Status of myocardial antioxidants in ischemia-reperfusion injury. *Cardiovascular Research*.

[28] Luan H.-F., Zhao Z.-B., Zhao Q.-H., Zhu P., Xiu M.-Y., Ji Y. (2012). Hydrogen sulfide postconditioning protects isolated rat hearts against ischemia and reperfusion injury mediated by the JAK2/STAT3 survival pathway. *Brazilian Journal of Medical and Biological Research*.

[29] Jin H. F., Wang Y., Wang X. B., Sun Y., Tang C. S., Du J. B. (2013). Sulfur dioxide preconditioning increases antioxidative capacity in rat with myocardial ischemia reperfusion (I/R) injury. *Nitric Oxide: Biology and Chemistry*.

[30] Osipov R. M., Robich M. P., Feng J. (2009). Effect of hydrogen sulfide in a porcine model of myocardial ischemia-reperfusion: comparison of different administration regimens and characterization of the cellular mechanisms of protection. *Journal of Cardiovascular Pharmacology*.

[31] Shymans'ka T. V., Hoshovs'ka I. V., Semenikhina O. M., Sahach V. F. (2012). Effect of hydrogen sulfide on isolated rat heart reaction under volume load and ischemia-reperfusion. *Fiziolohichnyǐ Zhurnal*.

[32] Zhuo Y., Chen P. F., Zhang A. Z., Zhong H., Chen C. Q., Zhu Y. Z. (2009). Cardioprotective effect of hydrogen sulfide in ischemic reperfusion experimental rats and its influence on expression of survivin gene. *Biological and Pharmaceutical Bulletin*.

[33] Elrod J. W., Calvert J. W., Morrison J. (2007). Hydrogen sulfide attenuates myocardial ischemia-reperfusion injury by preservation of mitochondrial function. *Proceedings of the National Academy of Sciences of the United States of America*.

[34] Olivetti G., Quaini F., Sala R. (1996). Acute myocardial infarction in humans is associated with activation of programmed myocyte cell death in the surviving portion of the heart. *Journal of Molecular and Cellular Cardiology*.

[35] Anversa P., Cheng W., Liu Y., Leri A., Redaelli G., Kajstura J. (1998). Apoptosis and myocardial infarction. *Basic Research in Cardiology*.

[36] Zhu Y. Z., Zhong J. W., Ho P. (2007). Hydrogen sulfide and its possible roles in myocardial ischemia in experimental rats. *Journal of Applied Physiology*.

[37] Wang Q., Wang X.-L., Liu H.-R., Rose P., Zhu Y.-Z. (2010). Protective effects of cysteine analogues on acute myocardial ischemia: novel modulators of endogenous H_2_S production. *Antioxidants & Redox Signaling*.

[38] Shen Y., Shen Z., Miao L. (2014). MiRNA-30 family inhibition protects against cardiac ischemic injury by regulating cystathionine-gamma-lyase expression. *Antioxidants & Redox Signaling*.

[39] Qipshidze N., Metreveli N., Mishra P. K., Lominadze D., Tyagi S. C. (2012). Hydrogen sulfide mitigates cardiac remodeling during myocardial infarction via improvement of angiogenesis. *International Journal of Biological Sciences*.

[40] Xie X., Sun A., Zhu W. (2012). Transplantation of mesenchymal stem cells preconditioned with hydrogen sulfide enhances repair of myocardial infarction in rats. *The Tohoku Journal of Experimental Medicine*.

[41] Pourkhalili K., Hajizadeh S., Tiraihi T. (2009). Ischemia and reperfusion-induced arrhythmias: role of hyperoxic preconditioning. *Journal of Cardiovascular Medicine*.

[42] Zhang Z., Huang H., Liu P., Tang C., Wang J. (2007). Hydrogen sulfide contributes to cardioprotection during ischemia-reperfusion injury by opening KATP channels. *Canadian Journal of Physiology and Pharmacology*.

[43] Bian J.-S., Yong Q. C., Pan T.-T. (2006). Role of hydrogen sulfide in the cardioprotection caused by ischemic preconditioning in the rat heart and cardiac myocytes. *The Journal of Pharmacology and Experimental Therapeutics*.

[44] Roell W., Lewalter T., Sasse P. (2007). Engraftment of connexin 43-expressing cells prevents post-infarct arrhythmia. *Nature*.

[45] Huang J. L., Wang D. M., Zheng J. B., Huang X. S., Jin H. (2012). Hydrogen sulfide attenuates cardiac hypertrophy and fibrosis induced by abdominal aortic coarctation in rats. *Molecular Medicine Reports*.

[46] Yong Q. C., Pan T.-T., Hu L.-F., Bian J.-S. (2008). Negative regulation of *β*-adrenergic function by hydrogen sulphide in the rat hearts. *Journal of Molecular and Cellular Cardiology*.

[47] Qi G.-M., Jia L.-X., Li Y.-L., Li H.-H., Du J. (2014). Adiponectin suppresses angiotensin II-induced inflammation and cardiac fibrosis through activation of macrophage autophagy. *Endocrinology*.

[48] Camelliti P., Borg T. K., Kohl P. (2005). Structural and functional characterisation of cardiac fibroblasts. *Cardiovascular Research*.

[49] Mishra P. K., Tyagi N., Sen U., Givvimani S., Tyagi S. C. (2010). H_2_S ameliorates oxidative and proteolytic stresses and protects the heart against adverse remodeling in chronic heart failure. *American Journal of Physiology—Heart and Circulatory Physiology*.

[50] Li G.-R., Sun H.-Y., Chen J.-B., Zhou Y., Tse H.-F., Lau C.-P. (2009). Characterization of multiple ion channels in cultured human cardiac fibroblasts. *PLoS ONE*.

[51] Sheng J., Shim W., Wei H. (2013). Hydrogen sulphide suppresses human atrial fibroblast proliferation and transformation to myofibroblasts. *Journal of Cellular and Molecular Medicine*.

[52] Wang X., Wang Q., Guo W., Zhu Y. Z. (2011). Hydrogen sulfide attenuates cardiac dysfunction in a rat model of heart failure: a mechanism through cardiac mitochondrial protection. *Bioscience Reports*.

[53] Pan L.-L., Liu X.-H., Shen Y.-Q. (2013). Inhibition of NADPH oxidase 4-related signaling by sodium hydrosulfide attenuates myocardial fibrotic response. *International Journal of Cardiology*.

[54] Indolfi C., di Lorenzo E., Perrino C. (2002). Hydroxymethylglutaryl coenzyme a reductase inhibitor simvastatin prevents cardiac hypertrophy induced by pressure overload and inhibits p21*ras* activation. *Circulation*.

[55] Lu F., Xing J., Zhang X. (2013). Exogenous hydrogen sulfide prevents cardiomyocyte apoptosis from cardiac hypertrophy induced by isoproterenol. *Molecular and Cellular Biochemistry*.

[56] Nicholson C. K., Lambert J. P., Molkentin J. D., Sadoshima J., Calvert J. W. (2013). Thioredoxin 1 is essential for sodium sulfide-mediated cardioprotection in the setting of heart failure. *Arteriosclerosis, Thrombosis, and Vascular Biology*.

[57] Yang F., Liu Z., Wang Y., Li Z., Yu H., Wang Q. (2014). Hydrogen sulfide endothelin-induced myocardial hypertrophy in rats and the mechanism involved. *Cell Biochemistry and Biophysics*.

[58] Huang J., Wang D., Zheng J., Huang X., Jin H. (2012). Hydrogen sulfide attenuates cardiac hypertrophy and fibrosis induced by abdominal aortic coarctation in rats. *Molecular Medicine Reports*.

[59] Padiya R., Chowdhury D., Borkar R., Srinivas R., Bhadra M. P., Banerjee S. K. (2014). Garlic attenuates cardiac oxidative stress via activation of PI3K/AKT/Nrf2-Keap1 pathway in fructose-fed diabetic rat. *PLoS ONE*.

[60] Calvert J. W., Elston M., Nicholson C. K. (2010). Genetic and pharmacologic hydrogen sulfide therapy attenuates ischemia-induced heart failure in mice. *Circulation*.

[61] Kondo K., Bhushan S., King A. L. (2013). H2S protects against pressure overload-induced heart failure via upregulation of endothelial nitric oxide synthase. *Circulation*.

[62] Kondo K., Bhushan S., Condit M. E., King A. L., Predmore B. L., Lefer d. J. (2011). Hydrogen sulfide attenuates cardiac dysfunction following pressure overload induced hypertrophy and heart failure via augmentation of angiogenesis. *Circulation*.

[63] Polhemus D. J., Kondo K., Bhushan S. (2013). Hydrogen sulfide attenuates cardiac dysfunction after heart failure via induction of angiogenesis. *Circulation: Heart Failure*.

[64] Givvimani S., Kundu S., Narayanan N. (2013). TIMP-2 mutant decreases MMP-2 activity and augments pressure overload induced LV dysfunction and heart failure. *Archives of Physiology and Biochemistry*.

[65] Zhang C. Y., Li X. H., Zhang T., Fu J., Cui X. D. (2013). Hydrogen sulfide upregulates heme oxygenase-1 expression in rats with volume overload-induced heart failure. *Biomedical Reports*.

[66] Liu Y.-H., Lu M., Xie Z.-Z. (2014). Hydrogen sulfide prevents heart failure development via inhibition of renin release from mast cells in isoproterenol-treated rats. *Antioxidants & Redox Signaling*.

[67] Huang C., Kan J., Liu X. (2013). Cardioprotective effects of a novel hydrogen sulfide agent-controlled release formulation of S-propargyl-cysteine on heart failure rats and molecular mechanisms. *PLoS ONE*.

[68] Kan J. T., Guo W., Huang C. R., Bao G. Z., Zhu Y. C., Zhu Y. Z. (2014). S-propargyl-cysteine, a novel water-soluble modulator of endogenous hydrogen sulfide, promotes angiogenesis through activation of signal transducer and activator of transcription 3. *Antioxidants & Redox Signaling*.

[69] Asghar O., Al-Sunni A., Khavandi K. (2009). Diabetic cardiomyopathy. *Clinical Science*.

[70] Dutta M., Biswas U. K., Chakraborty R., Banerjee P., Raychaudhuri U., Kumar A. (2014). Evaluation of plasma H_2_S levels and H_2_S synthesis in streptozotocin induced Type-2 diabetes-an experimental study based on Swietenia macrophylla seeds. *Asian Pacific Journal of Tropical Biomedicine*.

[71] Jain S. K., Bull R., Rains J. L. (2010). Low levels of hydrogen sulfide in the blood of diabetes patients and streptozotocin-treated rats causes vascular inflammation?. *Antioxidants & Redox Signaling*.

[72] Xu W., Wu W., Chen J. (2013). Exogenous hydrogen sulfide protects H9c2 cardiac cells against high glucose-induced injury by inhibiting the activities of the p38 MAPK and ERK1/2 pathways. *International Journal of Molecular Medicine*.

[73] Wei W.-B., Hu X., Zhuang X.-D., Liao L.-Z., Li W.-D. (2014). GYY4137, a novel hydrogen sulfide-releasing molecule, likely protects against high glucose-induced cytotoxicity by activation of the AMPK/mTOR signal pathway in H9c2 cells. *Molecular and Cellular Biochemistry*.

[74] Zhou X., An G., Lu X. (2015). Hydrogen sulfide attenuates the development of diabetic cardiomyopathy. *Clinical Science*.

[75] Peake B. F., Nicholson C. K., Lambert J. P. (2013). Hydrogen sulfide preconditions the db/db diabetic mouse heart against ischemia-reperfusion injury by activating Nrf2 signaling in an Erk-dependent manner. *The American Journal of Physiology—Heart and Circulatory Physiology*.

[76] Sun W.-H., Liu F., Chen Y., Zhu Y.-C. (2012). Hydrogen sulfide decreases the levels of ROS by inhibiting mitochondrial complex IV and increasing SOD activities in cardiomyocytes under ischemia/reperfusion. *Biochemical and Biophysical Research Communications*.

[77] Szabõ C. (2007). Hydrogen sulphide and its therapeutic potential. *Nature Reviews Drug Discovery*.

[78] Calvert J. W., Coetzee W. A., Lefer D. J. (2010). Novel insights into hydrogen sulfide-mediated cytoprotection. *Antioxidants & Redox Signaling*.

[79] Pan T. T., Neo K. L., Hu L. F., Yong Q. C., Bian J. S. (2008). H2S preconditioning-induced PKC activation regulates intracellular calcium handling in rat cardiomyocytes. *The American Journal of Physiology—Cell Physiology*.

[80] Muellner M. K., Schreier S. M., Laggner H. (2009). Hydrogen sulfide destroys lipid hydroperoxides in oxidized LDL. *Biochemical Journal*.

[81] Marí M., Morales A., Colell A., García-Ruiz C., Fernández-Checa J. C. (2009). Mitochondrial glutathione, a key survival antioxidant. *Antioxidants & Redox Signaling*.

[82] Alves M. G., Soares A. F., Carvalho R. A., Oliveira P. J. (2011). Sodium hydrosulfide improves the protective potential of the cardioplegic histidine buffer solution. *European Journal of Pharmacology*.

[83] Aon M. A., Cortassa S., Akar F. G., O'Rourke B. (2006). Mitochondrial criticality: a new concept at the turning point of life or death. *Biochimica et Biophysica Acta—Molecular Basis of Disease*.

[84] Churchill E. N., Mochly-Rosen D. (2007). The roles of PKC*δ* and *ε* isoenzymes in the regulation of myocardial ischaemia/reperfusion injury. *Biochemical Society Transactions*.

[85] Murphy E., Steenbergen C. (2007). Preconditioning: the mitochondrial connection. *Annual Review of Physiology*.

[86] Zhou X., Lu X. (2013). Hydrogen sulfide inhibits high-glucose-induced apoptosis in neonatal rat cardiomyocytes. *Experimental Biology and Medicine*.

[87] Sodha N. R., Clements R. T., Feng J. (2008). The effects of therapeutic sulfide on myocardial apoptosis in response to ischemia-reperfusion injury. *European Journal of Cardio-Thoracic Surgery*.

[88] Yao L.-L., Huang X.-W., Wang Y.-G., Cao Y.-X., Zhang C.-C., Zhu Y.-C. (2010). Hydrogen sulfide protects cardiomyocytes from hypoxia/reoxygenation-induced apoptosis by preventing GSK-3*β*-dependent opening of mPTP. *American Journal of Physiology—Heart and Circulatory Physiology*.

[89] Pomerantz B. J., Reznikov L. L., Harken A. H., Dinarello C. A. (2001). Inhibition of caspase 1 reduces human myocardial ischemic dysfunction via inhibition of IL-18 and IL-1 beta. *Proceedings of the National Academy of Sciences of the United States of America*.

[90] Hennein H. A., Ebba H., Rodriguez J. L. (1994). Relationship of the proinflammatory cytokines to myocardial ischemia and dysfunction after uncomplicated coronary revascularization. *Journal of Thoracic and Cardiovascular Surgery*.

[91] Dinarello C. A. (2000). Proinflammatory cytokines. *Chest*.

[92] Whiteman M., Winyard P. G. (2011). Hydrogen sulfide and inflammation: the good, the bad, the ugly and the promising. *Expert Review of Clinical Pharmacology*.

[93] Sodha N. R., Clements R. T., Feng J. (2009). Hydrogen sulfide therapy attenuates the inflammatory response in a porcine model of myocardial ischemia/reperfusion injury. *The Journal of Thoracic and Cardiovascular Surgery*.

[94] Zanardo R. C. O., Brancaleone V., Distrutti E., Fiorucci S., Cirino G., Wallace J. L. (2006). Hydrogen sulfide is an endogenous modulator of leukocyte-mediated inflammation. *The FASEB Journal*.

[95] Pan L.-L., Liu X.-H., Gong Q.-H., Zhu Y.-Z. (2011). S-Propargyl-cysteine (SPRC) attenuated lipopolysaccharide-induced inflammatory response in H9c2 cells involved in a hydrogen sulfide-dependent mechanism. *Amino Acids*.

[96] Szabó C., Papapetropoulos A. (2011). Hydrogen sulphide and angiogenesis: mechanisms and applications. *British Journal of Pharmacology*.

[97] Papapetropoulos A., Pyriochou A., Altaany Z. (2009). Hydrogen sulfide is an endogenous stimulator of angiogenesis. *Proceedings of the National Academy of Sciences of the United States of America*.

[98] Givvimani S., Munjal C., Gargoum R. (2011). Hydrogen sulfide mitigates transition from compensatory hypertrophy to heart failure. *Journal of Applied Physiology*.

[99] Zhao W. M., Zhang J., Lu Y. J., Wang R. (2001). The vasorelaxant effect of H_2_S as a novel endogenous gaseous K_ATP_ channel opener. *The EMBO Journal*.

[100] Kai S., Tanaka T., Daijo H. (2012). Hydrogen sulfide inhibits hypoxia-but not anoxia-induced hypoxia-inducible factor 1 activation in a von hippel-lindau-and mitochondria-dependent manner. *Antioxidants and Redox Signaling*.

[101] Tao B. B., Liu S. Y., Zhang C. C. (2013). VEGFR2 functions as an H_2_S-targeting receptor protein kinase with its novel Cys1045–Cys1024 disulfide bond serving as a specific molecular switch for hydrogen sulfide actions in vascular endothelial cells. *Antioxidants & Redox Signaling*.

[102] Bers D. M. (2008). Calcium cycling and signaling in cardiac myocytes. *Annual Review of Physiology*.

[103] Tang G. H., Wu L. Y., Wang R. (2010). Interaction of hydrogen sulfide with ion channels. *Clinical and Experimental Pharmacology and Physiology*.

[104] Sun Y. G., Cao Y. X., Wang W. W., Ma S. F., Yao T., Zhu Y. C. (2008). Hydrogen sulphide is an inhibitor of L-type calcium channels and mechanical contraction in rat cardiomyocytes. *Cardiovascular Research*.

[105] Zhang R. Y., Sun Y., Tsai H. J., Tang C. S., Jin H. F., Du J. B. (2012). Hydrogen sulfide inhibits L-type calcium currents depending upon the protein sulfhydryl state in rat cardiomyocytes. *PLoS ONE*.

[106] Vassort G., Talavera K., Alvarez J. L. (2006). Role of T-type Ca^2+^ channels in the heart. *Cell Calcium*.

[107] Elies J., Scragg J. L., Huang S. (2014). Hydrogen sulfide inhibits Cav3.2 T-type Ca^2+^ channels. *The FASEB Journal*.

[108] Tang G., Wu L., Liang W., Wang R. (2005). Direct stimulation of K_ATP_ channels by exogenous and endogenous hydrogen sulfide in vascular smooth muscle cells. *Molecular Pharmacology*.

[109] Lee S. W., Cheng Y., Moore P. K., Bian J. S. (2007). Hydrogen sulphide regulates intracellular pH in vascular smooth muscle cells. *Biochemical and Biophysical Research Communications*.

[110] Johansen D., Ytrehus K., Baxter G. F. (2006). Exogenous hydrogen sulfide (H_2_S) protects against regional myocardial ischemia-reperfusion injury—evidence for a role of K_ATP_ channels. *Basic Research in Cardiology*.

[111] Zhang Z., Huang H., Liu P., Tang C., Wang J. (2007). Hydrogen sulfide contributes to cardioprotection during ischemia-reperfusion injury by opening K_ATP_ channels. *Canadian Journal of Physiology and Pharmacology*.

[112] Strege P. R., Bernard C. E., Kraichely R. E. (2011). Hydrogen sulfide is a partially redox-independent activator of the human jejunum Na^+^ channel, NA_v_1.5. *The American Journal of Physiology—Gastrointestinal and Liver Physiology*.

[113] Malekova L., Krizanova O., Ondrias K. (2009). H_2_S and HS^−^ donor NaHS inhibits intracellular chloride channels. *General Physiology and Biophysics*.

[114] King A. L., Polhemus D. J., Bhushan S. (2014). Hydrogen sulfide cytoprotective signaling is endothelial nitric oxide synthase-nitric oxide dependent. *Proceedings of the National Academy of Sciences of the United States of America*.

[115] Whiteman M., Li L., Kostetski I. (2006). Evidence for the formation of a novel nitrosothiol from the gaseous mediators nitric oxide and hydrogen sulphide. *Biochemical and Biophysical Research Communications*.

[116] Predmore B. L., Kondo K., Bhushan S. (2012). The polysulfide diallyl trisulfide protects the ischemic myocardium by preservation of endogenous hydrogen sulfide and increasing nitric oxide bioavailability. *The American Journal of Physiology—Heart and Circulatory Physiology*.

[117] Łowicka E., Bełtowski J. (2007). Hydrogen sulfide (H_2_S)—the third gas of interest for pharmacologists. *Pharmacological Reports*.

[118] Fiedler J., Batkai S., Thum T. (2014). MicroRNA-based therapy in cardiology. *Herz*.

[119] Shen J., Xing T., Yuan H. (2013). Hydrogen sulfide improves drought tolerance in *Arabidopsis thaliana* by microRNA expressions. *PLoS ONE*.

[120] Huang X., Gao Y., Qin J., Lu S. (2014). The role of miR-34a in the hepatoprotective effect of hydrogen sulfide on ischemia/reperfusion injury in young and old rats. *PLoS ONE*.

[121] Liu J., Hao D.-D., Zhang J.-S., Zhu Y.-C. (2011). Hydrogen sulphide inhibits cardiomyocyte hypertrophy by up-regulating miR-133a. *Biochemical and Biophysical Research Communications*.

[122] Toldo S., Das A., Mezzaroma E. (2014). Induction of microRNA-21 with exogenous hydrogen sulfide attenuates myocardial ischemic and inflammatory injury in mice. *Circulation: Cardiovascular Genetics*.

[123] Kimura Y., Kimura H. (2004). Hydrogen sulfide protects neurons from oxidative stress. *The FASEB Journal*.

[124] Caliendo G., Cirino G., Santagada V., Wallace J. L. (2010). Synthesis and biological effects of hydrogen sulfide (H_2_S): development of H_2_S-releasing drugs as pharmaceuticals. *Journal of Medicinal Chemistry*.

[125] Zhao Y., Bhushan S., Yang C. (2013). Controllable hydrogen sulfide donors and their activity against myocardial ischemia-reperfusion injury. *ACS Chemical Biology*.

[126] Predmore B. L., Kondo K., Bhushan S. (2012). The polysulfide diallyl trisulfide protects the ischemic myocardium by preservation of endogenous hydrogen sulfide and increasing nitric oxide bioavailability. *American Journal of Physiology—Heart and Circulatory Physiology*.

[127] Tsai C.-Y., Wang C.-C., Lai T.-Y. (2013). Antioxidant effects of diallyl trisulfide on high glucose-induced apoptosis are mediated by the PI3K/Akt-dependent activation of Nrf2 in cardiomyocytes. *International Journal of Cardiology*.

[128] Lei Y. P., Liu C. T., Sheen L. Y., Chen H. W., Lii C. K. (2010). Diallyl disulfide and diallyl trisulfide protect endothelial nitric oxide synthase against damage by oxidized low-density lipoprotein. *Molecular Nutrition and Food Research*.

[129] Shin C. C., Moore P. K., Zhu Y. Z. (2007). S-allylcysteine mediates cardioprotection in an acute myocardial infarction rat model via a hydrogen sulfide-mediated pathway. *The American Journal of Physiology—Heart and Circulatory Physiology*.

[130] Wang Q., Liu H.-R., Mu Q., Rose P., Zhu Y. Z. (2009). S-propargyl-cysteine protects both adult rat hearts and neonatal cardiomyocytes from ischemia/hypoxia injury: the contribution of the hydrogen sulfide-mediated pathway. *Journal of Cardiovascular Pharmacology*.

